# The Shared Crosstalk of Multiple Pathways Involved in the Inflammation between Rheumatoid Arthritis and Coronary Artery Disease Based on a Digital Gene Expression Profile

**DOI:** 10.1371/journal.pone.0113659

**Published:** 2014-12-16

**Authors:** Xuyan Niu, Cheng Lu, Cheng Xiao, Zhiguo Zhang, Miao Jiang, Dan He, Yanqin Bian, Ge Zhang, Zhaoxiang Bian, Aiping Lu

**Affiliations:** 1 Institute of Basic Research in Clinical Medicine, China Academy of Chinese Medical Sciences, Beijing, 100700, China; 2 China-Japan Friendship Hospital, Beijing, 100029, China; 3 Institute of Basic Theory, China Academy of Chinese Medical Sciences, Beijing, 100700, China; 4 E-Institute of Chinese Traditional Internal Medicine, Shanghai Municipal Education Commission, Shanghai, 201203, China; 5 Institute for Advancing Translational Medicine in Bone & Joint Diseases, School of Chinese Medicine, Hong Kong Baptist University, Hong Kong, China; Washington Hospital Center, United States of America

## Abstract

Rheumatoid arthritis (RA) and coronary artery disease (CAD) are both complex inflammatory diseases, and an increased prevalence of CAD and a high rate of mortality have been observed in RA patients. But the molecular mechanism of inflammation that is shared between the two disorders is unclear. High-throughput techniques, such as transcriptome analysis, are becoming important tools for genetic biomarker discovery in highly complex biological samples, which is critical for the diagnosis, prognosis, and treatment of disease. In the present study, we reported one type of transcriptome analysis method: digital gene expression profiling of peripheral blood mononuclear cells of 10 RA patients, 10 CAD patients and 10 healthy people. In all, 213 and 152 differently expressed genes (DEGs) were identified in RA patients compared with normal controls (RA *vs.* normal) and CAD patients compared with normal controls (CAD *vs.* normal), respectively, with 73 shared DEGs between them. Using this technique in combination with Ingenuity Pathways Analysis software, the effects on inflammation of four shared canonical pathways, three shared activated predicted upstream regulators and three shared molecular interaction networks were identified and explored. These shared molecular mechanisms may provide the genetic basis and potential targets for optimizing the application of current drugs to more effectively treat these diseases simultaneously and for preventing one when the other is diagnosed.

## Introduction

Rheumatoid arthritis (RA) is a systemic, chronic and progressive inflammatory disease that affects mainly the synovial membrane of joints [1]. Systemic inflammatory diseases are associated with increased coronary artery disease (CAD) morbidity and mortality [2]. RA and CAD are both complex inflammatory diseases, and an increased prevalence of CAD and a high rate of mortality were observed in RA patients [3]. In addition, the relative risk of a myocardial infarction is three-fold higher among women with RA compared to controls [4]. Excessive cardiovascular events observed in individuals with RA are not fully explained by the traditional risk factors [5]–[7], and the underlying mechanism behind the high prevalence of CAD morbidity in RA is not completely understood [8].

There is an increasing interest in identifying ‘nontraditional’ novel risk factors such as genetic polymorphisms in the study of the molecular mechanisms of complex diseases [9], [10]. It has been speculated that genes that play an important role in the development and progression of RA may also play a role in comorbidities and mortality in this disease [11]. In clinical research, specific disease-modifying drugs (e.g., methotrexate and tumor necrosis factor (TNF) inhibitors) effectively control inflammation and frequently used in RA but not in prevention of CAD, also reduce CAD risk but with many limitations such as side effects, limited targets and absence of studies on effects and safety in depth [12]. For example, current clinical evidences suggested that abatacept, a novel CD80/86-CD28 T cell co-stimulation modulator, was effective on reducing inflammatory biomarkers in RA patients [13]. Coincidentally, it had also reported T-cell CD80/86-CD28 co-stimulation was vital for post-interventional accelerated atherosclerosis development, indicating promising clinical potential for prevention of post-interventional remodeling in CAD by abatacept [14]. Patrick H. Dessein *et al.* proved interleukin-6 (IL-6) concentrations independently contribute markedly to endothelial activation in RA more than other cardiovascular risk factors, which enlightened assessment of IL-6 concentrations might enhance cardiovascular risk stratification in RA [15]. Thus the shared novel molecular factors and pathways in RA and CAD may be considered new therapeutic or predicted targets [16], [17]. We therefore hypothesized that lots of commonly shared genes, molecules and pathways involved in chronic inflammation may exist in the form of networks both in RA and CAD and may serve as potential treatment targets.

Recently, several high-throughput techniques have been developed to study the expression of mRNAs, proteins, and metabolites [18], such as the next-generation sequencing (NGS) platform [19]–[21]. Currently, one NGS protocol, 3′- tag digital gene expression (DGE) developed by Illumina (Illumina Inc., San Diego, CA, USA), has been widely used in transcriptome studies [22], [23]. Some reports have focused on the molecular mechanisms of pathophysiologic changes during RA or CAD independently by transcriptome or gene expression profiles technology [23]–[25], but few studies have focused on the associations and shared genes between RA and CAD by high-throughput techniques such as transcriptome analysis.

In the present study, we preformed the DGE method on peripheral blood mononuclear cells (PBMCs) of RA and CAD patients to review the transcriptome changes between them by identifying the DEGs compared with healthy volunteers. We analyzed the associations of pathways and networks related with those genes identified by Ingenuity Pathways Analysis (IPA, http://www.ingenuity.com) [26]. This research may provide the genetic basis for the shared prevention and treatment of these two complex related diseases, utilizing the inflammatory mechanisms to optimize the application of currently available drugs to more effectively treat them simultaneously.

## Methods

### Patients

RA and CAD patients and healthy volunteers were recruited at China-Japan Friendship Hospital in Beijing. The active RA sufferers were selected via their general practitioner from the outpatient rheumatology clinic, and their diagnosis was defined by the 1987 American College of Rheumatology revised criteria and the 2010 American College of Rheumatology/European League Against Rheumatism classification criteria for RA [27], [28]. Disease activity was assessed by the Disease Activity Score in 28 joints (DAS28) [29]. For the purposes of this study, the RA patients were considered to have active RA if they displayed the following symptoms for greater than 6 months: 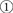 morning stiffness for more than 30 min; 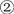 swelling in 3 or more joints, swelling of finger and/or wrist joints, symmetric swelling, rheumatoid nodules, positive rheumatoid factor, or radiographic erosions in the hand and/or wrist; 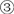 6 or more tender joints; 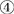 DAS28>3.2 [29]; 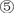 erythrocyte sedimentation rate (ESR)≥28 mm/h or C reactive protein (CRP) 20% higher than the normal value [27]. Because RA is two to three times more common in females than males, we chose female patients as the observed subjects in this study [30].

The CAD patients were diagnosed using diagnostic coronary arteriography to detect flow-limiting coronary artery stenosis, which was identified based on electrocardiogram (ECG) results (the inclusion criteria was shown below in detail) and adjudicated by two trained physicians on the basis of clinical examination indexes. Considering that the inflammatory response in CAD patients with acute coronary syndrome (ACS) is more obvious than in patients with stable angina [31], we chose CAD patients with ACS for inclusion in this study. ACS is a broad term that includes unstable angina (UA), non-ST elevation myocardial infarction (NSTEMI) and ST elevation myocardial infarction (STEMI). Cases of ACS were determined by both formal (World Health Organization 1984 criteria for acute myocardial infarction (AMI), Braunwald criteria for unstable angina (UA)) and medical record criteria. Myocardial infarction (MI) was defined by the presence of symptoms consistent with cardiac ischemia within 24 hours of hospital presentation and troponin I levels above the 99^th^ percentile with a test-specific coefficient of variation <10% [32]. STEMI was defined by the presence of criteria for MI plus one of the following: (a) persistent ST segment elevation of ≥1 mm in two contiguous ECG leads or (b) the presence of a new or presumably new left bundle branch block. NSTEMI was defined by the presence of criteria for MI but not for STEMI. The UA diagnosis required the presence of symptoms consistent with cardiac ischemia 24 hours prior to hospital admission, absence of MI criteria and at least one of the following: (a) history of coronary heart disease (CHD); (b) positive coronary disease stratification test (invasive or noninvasive); (c) transient ST segment changes ≥0.5 mm in two contiguous leads, new T-wave inversion of ≥1 mm and/or pseudonormalization of previously inverted T waves; (d) troponin>0.05 ng/mL; or (e) diagnostic concordance of two independent doctors.

Exclusion criteria included current or prior treatment with prednisone, systolic blood pressure <90 or>150 mm Hg, heart rate <50 or>130 bpm, diabetes mellitus, dyslipidemia, or a history of smoking. Patients with severe diseases of the lungs, liver, kidneys, and patients with mental or blood system disturbances, as well as women who were pregnant, breast-feeding or planning to become pregnant in the next 8 months were excluded from the study. The RA patients included were not diagnosed with any type of CAD, and the CAD patients did not have a concurrent RA diagnosis. The participants in the normal control group were considered healthy if their medical history did not reveal any chronic diseases, endemic infectious diseases or autoimmune diseases, and their physical examination and blood tests failed to show otherwise.

Peripheral blood samples were collected from 10 female RA patients, 10 female CAD patients, and 10 female healthy controls. And the detailed baseline characters of all participants are shown in **Table 1**. Age- and sex-matched blood samples from healthy individuals were selected as the healthy controls. Peripheral blood samples of 3 mL were collected in the morning before breakfast from each individual participant in this study. The PBMCs were extracted immediately from each blood sample and then stored in Trizol under −80°C until analysis. All protocols involving human subjects were approved by the ethics committee of China-Japan Friendship Hospital (ethics ID: 2014–58), and informed consents were signed by all participants before commencing the study.

**Table 1:** Baseline characteristics of the RA, CAD patients and normal groups.

Characteristic	Normal	RA	CAD
**Female Cases**	10	10	10
**Age, Mean years ±SD**	45.8±3.5	54.5±7.1	59.2±4.2
**BMI (kg/m^2^), mean ±SD**	22.34±1.83	25.81±2.62	24.43±3.02
**ESR (mm/h), mean ±SD**	/	39.14±29.53	/
**CRP (mg/L), mean ±SD**	/	15.48±15.40	/
**RF (IU/mL), mean ±SD**	/	63.76±71.81	/
**DAS28, mean ±SD**	/	4.62±0.52	/
**Troponin I (TnI) (ng/mL), (cases in CAD group<0.05 ng/mL, cases cases in CAD group>0.05 ng/mL)**	/	/	5, 5
**CK-MB (ng/mL),(cases in CAD group <1.0 ng/mL, cases in CAD group>1.0 ng/mL)**	/	/	5, 5

### PBMC isolation and total RNA extraction

PBMCs were isolated by density gradient centrifugation over Ficoll-Hypaque gradient solution (Histopaque-1077, Sigma-Aldrich, USA), according to the manufacturer's instructions. Heparinized whole blood (3 mL) was diluted to 6 mL with phosphate buffered saline (PBS, pH 7.4), layered on top of 3 mL Histopaque and centrifuged for 30 min at 400×g. PBMCs were aspirated, washed twice, suspended in PBS and counted with a hemocytometer. PBMCs were lysed in Trizol reagent (1 mL/1×10^7^ PBMC) (Invitrogen, Karlsruhe, Germany; Carlsbad, CA) and stored at −80°C until further processing.

PBMC samples from RA, CAD patients and healthy normal controls were selected to isolate total RNA. Total RNA was isolated by the Trizol extraction method according to the manufacturer's protocol. Total RNA was quantified with a NanoDrop ND-1000 spectrophotometer (Thermo Fisher Scientific Inc., Marietta, OH, USA) followed by quality assessment with the 2100 Bioanalyzer using an RNA 6000 LabChip kit (Agilent Technologies, Palo Alto, CA) according to the manufacturer's instructions. Acceptable quality values were in the 1.8–2.2 range for A260/A280 ratios, and for each total RNA sample, the RNA Integrity Number was>7.0.

### DGE tag profiling and identification of differentially expressed genes

For each sample, 4 µg of total RNA was purified by adsorption of biotin oligo magnetic beads. After the binding of mRNA, cDNA synthesis was performed. Double strand cDNA was introduced into the cDNA fragment digested by *NlaIII* endonuclease and the binging fragments containing sequences of CATG sites and adjacent poly A tails in the 3′ end. After the precipitation of the 3′ cDNA fragment, an Illumina adaptor 1 was added to the 5′ end. Both the adaptor 1 and CATG sites can be recognized by *MmeI*, which cuts at a downstream CATG site and produces fragments of 17 bp tags with adaptor 1. Adaptor 2 was added to the 3′ end of these tags after eliminating the fragment with beads in the 3′ end. Next, these sequences were prepared for Solexa sequencing.

Clean-tags were obtained by filtering the adaptor sequences and removing low-quality sequences (containing ambiguous bases). Only the tags with perfect matches or one mismatch were further considered and annotated based on the reference genes. The expression level of each gene was estimated by the frequency of clean tags and then normalized to TPM (number of transcripts per million clean tags) [22], which is a standard method and extensively used in DGE analysis [33]. TPM indicates reads per kilobase of transcript per million sequenced reads. The expression level of each gene was measured by the normalized number of matched clean tags.

The number of tags mapped to a given gene was considered to represent the expression level of this gene. Expression levels of a gene from two distinct samples were compared to give an expression difference. We identified the differentially expressed genes (DEGs) in the RA *vs*. the normal control group and the CAD *vs*. the normal control groups. Significance values for differences in expression were determined using a modified exact test, similar to *Fisher's exact* test. The *P*-values were adjusted using a *Benjamini* and Hochberg false discovery rate (FDR) of 1%. We classified the gene as differentially expressed only when the expression difference was more than 1.2-fold with *P*-value <0.01.

### Pathway and network analysis by IPA

The Gene Bank accession numbers and matched identified DEGs in RA *vs*. normal and CAD *vs*. normal groups were set up as the identifiers of the two datasets. Each dataset was saved as an Excel file and uploaded into the Ingenuity Pathways Analysis system (IPA, Ingenuity Systems, http://www.ingenuity.com), which enabled the discovery, visualization and exploration of molecular interactions to identify the biological mechanisms, pathways and functions most relevant to the experimental datasets, genes, or proteins of interest. The proof-of-knowledge based IPA was performed to characterize the biomarkers confirmed by the pattern recognition analyses to evaluate the biomarkers based on their metabolic or signaling associations in canonical pathways and biological networks [34]. We utilized the IPA analysis system with the ‘Core analysis’ platform to analyze the identified DEGs of RA *vs*. normal control and CAD *vs*. normal control groups; comparisons between the canonical pathways and biological networks between the two groups were also carried out using the ‘Comparison’ platform in IPA. In this study, the score was -10 logarithms of *Fisher's exact* test *p*-values in canonical pathway analysis by IPA. Significances for biological functions were then assigned to each network by determining a *p*-value for the enrichment of the genes in the network for such functions compared with the whole Ingenuity Pathway Knowledge Base as a reference set.

## Results

### Gene expression profiles in RA *vs*. normal and CAD *vs*. normal groups

**Figure 1:**
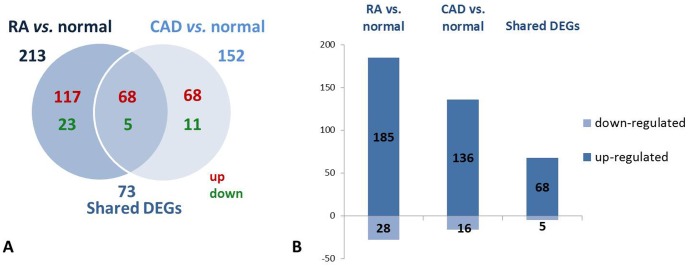
Gene expression in groups of RA *vs.* normal, CAD *vs.* normal and shared DEGs. A: Venn diagram showing the number of uniquely up-regulated (red) or down-regulated (green) genes comparing individuals with RA and CAD to normal persons and shared DEGs; B: Bar diagram indicating number of DEGs in the RA *vs.* normal and the CAD *vs.* normal groups and the shared DEGs between them.

Differentially expressed genes (DEGs) in the RA or CAD groups compared with the normal control group were determined (>1.2-fold change, false discovery rate (FDR) <0.05; **Tables S1, S2 in** **S1 File**). The number of DEGs is summarized in the Venn diagram (**Fig. 1A**) and the histogram (**Fig. 1B**). In comparison with the normal group, 213 and 152 DEGs were identified in the RA and CAD patients, respectively. It was striking that the majority of DEGs were up-regulated in the diseased states, both in RA and CAD. While 117 and 68 unique genes were up-regulated in RA and CAD, respectively, there were 23 and 11 significantly down-regulated genes in those two groups, respectively. **Figures 1A, B** show 73 shared DEGs between two states: 68 up-regulated and 5 down-regulated DEGs in both RA and CAD patients compared with normal controls. We also found the 73 shared DEGs in the two groups regulated with the same directions, respectively, compared to normal (**Table S3 in** **S1 File**).

### Functions annotation comparison of DEGs in RA and CAD showing inflammation related functions shared in top

**Figure 2:**
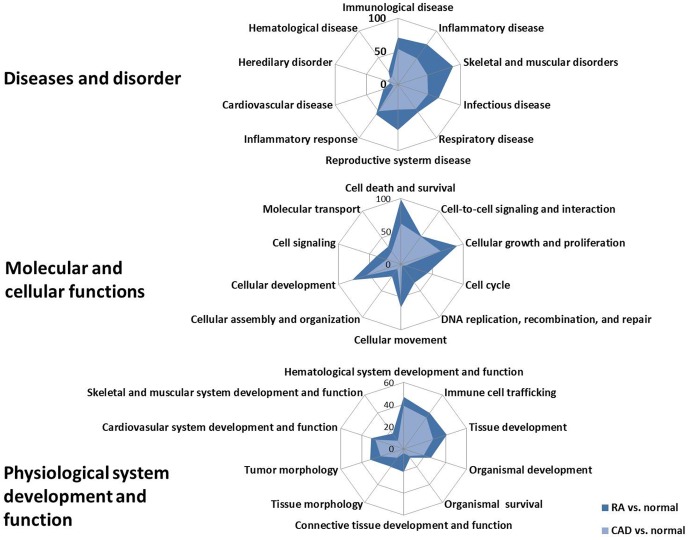
The shared functions of DEGs in individuals with RA and CAD compared to normal controls. Radar diagrams show the shared functions of DEGs in RA and CAD compared to normal controls in aspects of ‘Diseases and disorder’, ‘Molecular and cellular functions’, and ‘Physiological system development and function’. Blue areas represented the number of DEGs in RA *vs.* the normal number involved in the functions. Red areas represent the number of DEGs in CAD *vs.* normal controls involved in the functions.

IPA was used to identify functions that were significantly over-represented by the DEGs in the comparison of RA and CAD. Next, we classified counting the number of DEGs involved in functions in three aspects: ‘diseases and disorder’, ‘molecular and cellular functions’ and ‘physiological system development and function’, shown in **Fig. 2**. Within the three aspects, there were several shared functions of DEGs in both RA and CAD compared with the normal controls, especially at diseases and disorder functions; most of the shared functions were focused on the inflammation related functions, such as inflammatory diseases, infectious disease, and the inflammatory response. This phenomenon indicated that inflammatory processes play an important role in both RA and CAD disease development. At the same time, among the shared molecular and cellular functions of DEGs in both RA and CAD, the functions of cell death and survival, cellular growth and proliferation, and cellular development were more important. In addition, in the shared physiological system development and functions, there were more DEGs that participated in the hematological system development and function and immune cell trafficking in both RA and CAD. Thus, the most important shared functions in which most DEGs participated were inflammation related functions within the diseases and disorder functions.

### Network, function and canonical pathway analysis of shared DEGs in RA and CAD

**Figure 3:**
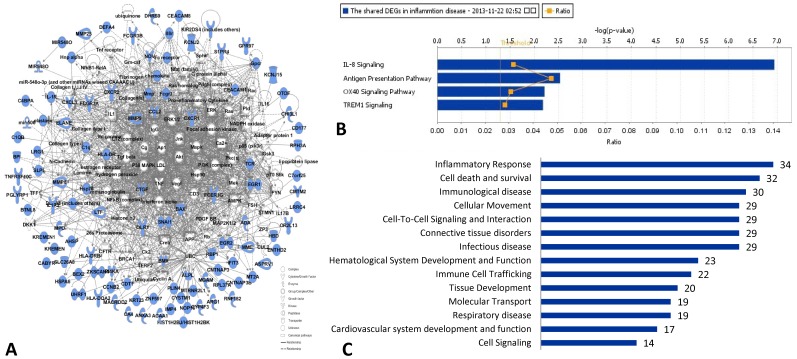
Network, molecular functions and canonical pathway analysis of shared DEGs in RA *vs.* normal and CAD *vs*. normal individuals. A: The molecular network of 95 shared DEGs in RA *vs.* normal and CAD *vs.* normal individuals; blue molecules represent the shared DEGs; B: the top 4 shared canonical pathways (with score (-log (*p*-value))>1.3, *p*-value of pathway <0.05) related with inflammation which 95 shared DEGs in RA vs. normal and CAD vs. normal involved in: IL-8 signaling; antigen presentation pathway; OX40 signaling pathway; TREM1 signaling; C: the function classification of shared DEGs in both RA *vs.* normal and CAD *vs.* normal, the number in the bar diagram represented the DEGs numbers participated in the corresponding pathways.

The shared molecular network was built up from 95 shared DEGs in both RA *vs.* normal controls and CAD *vs.* normal controls, as shown in **Fig. 3A**. The function annotations of the shared DEGs molecular network indicated inflammatory response was most important, involving the most shared DEGs: 34 shared DEGs participated in this function, as shown in **Fig. 3C**.

**Figure 4:**
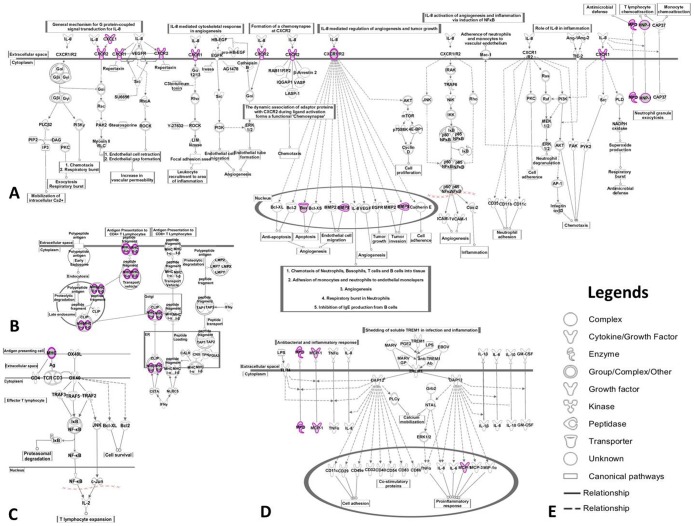
The significant commonly shared signaling canonical pathways between RA and CAD involved in inflammation. A: IL-8 signaling; B: antigen presentation pathway; C: OX40 signaling pathway; D: TREM1 signaling; E: picture legends. Purple circle: the same genes involved in the commonly shared pathways in RA and CAD.

Based on the shared DEGs in **Table S3 in** **S1 File**, we determined the top 4 shared biological pathways associated with inflammation in both RA and CAD using IPA (**Fig. 3B**). The calculation was either achieved according to the ratio (the number of genes from the data set that map to the canonical pathway in question divided by the total number of proteins that map to the same canonical pathway) or significance (*Fischer's* exact test was used to calculate a *p*-value determining the probability that the association between the proteins in the dataset and the canonical pathway was explained by chance alone). We chose the commonly shared pathways via score (-log (*p*-value))>1.3 (*p*-value of pathway <0.05) in DEGs of RA *vs*. normal controls and CAD *vs*. normal controls as significant shared pathways in which the DEGs of the two diseases were both involved, as shown in **Table 2**. Four shared signaling pathways related to inflammation between RA and CAD were found: IL-8 signaling, antigen presentation pathway, OX40 signaling pathway and TREM1 signaling. Additionally, 10 shared DEGs were also involved (**Table 2** **,** **Fig. 4**): CXCL1, CXCR1, CXCR2, BAX, DEFA1, MMP9, HLA-DRB4, HLA-DQA2, CCL2/MCP-1 and MPO, with same regulated directions. The shared DEGs play important roles in the 4 top shared signaling pathways and in the key nodes leading to inflammation.

**Table 2:** The top 4 shared signaling pathways related to inflammation and shared DEGs in both RA *vs*. normal and CAD *vs*. normal subjects.

No.	Shared pathways	Score of pathways (-log(*p*-value))	Shared DEGs involved in shared pathways	Fold of shared DEGs (-log(*p*-value))	
				RA	CAD
**1**	***IL-8 signaling***	6.94	BAX	1.39	1.98
			CXCL1	1.82	1.47
			CXCR1	1.96	1.62
			CXCR2	1.41	1.32
			DEFA1	1.85	2.47
			MMP9	1.90	2.26
			MPO	1.23	1.43
**2**	***Antigen presentation pathway***	2.53	HLA-DRB4	1.71	1.99
			HLA-DQA2	1.30	1.51
**3**	***OX40 signaling pathway***	2.21	HLA-DRB4	1.71	1.99
			HLA-DQA2	1.30	1.51
**4**	***TREM1 signaling***	2.18	CCL2	2.07	1.43
			MPO	1.23	1.43

### The shared associated networks between RA and CAD

**Figure 5:**
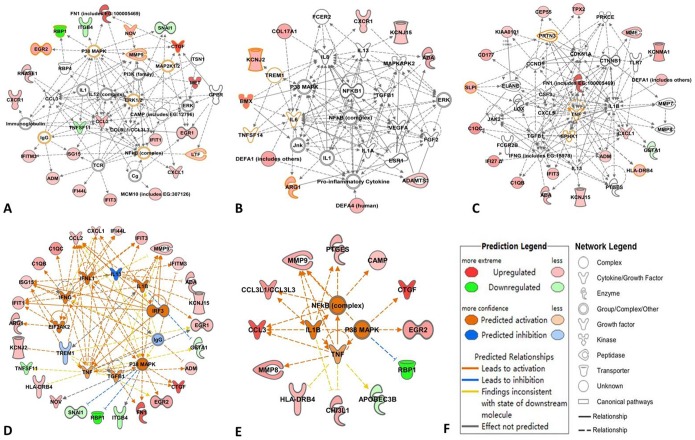
The shared molecular networks and upstream regulators networks of RA and CAD. A: shared associated network (SAN) -1; B: SAN-2; C: SAN-3; D: the network of upstream regulators and target molecules of DEGs in RA; E: the network of upstream regulators and target molecules of DEGs in CAD; F: the legends of predicted networks. In A, B and C, the molecules with orange circles stand for shared molecules between RA and CAD.

To further explore the commonly shared biological roots associated between RA and CAD, we identified molecular networks that were associated with DEGs in RA *vs*. normal controls and CAD *vs.* normal controls using IPA analysis. As shown in **Figs. 5A, 5B and 5C**, there were 3 shared associated networks (SANs) that were found between RA and CAD: shared associated network (SAN) -1, SAN-2 and SAN-3. The functions of the associated merged networks between RA and CAD are shown in **Table 3**. We found that the top functions of the three SANs were focused on immune cell trafficking, inflammatory response, inflammatory diseases, cell-to-cell signaling and interaction, and cell death and survival, most of which were related to biological processes of inflammation. Additionally, there were 11 shared DEGs involved in the three SANs: CTGF, EGR2, LTF, MMP9, NOV, RBP1, ARG, BMX, KCNJ2, HLA-DRB4, and SLPI.

**Table 3:** The top functions of shared associated networks (SANs) between RA and CAD and the shared molecules involved (The molecules in italics are shared DEGs in both RA *vs.* normal and CAD *vs.* normal groups).

No.	Shared molecules in shared associated network (SAN)	Top functions of shared associated networks
1	***CTGF***, ***EGR2***, ERK1/2, IgG, ***LTF***, MAP2K1/2, ***MMP9***, NF-κB complex, ***NOV***, p38 MAPK, ***RBP1***	Immune cell trafficking, inflammatory response, cell-to-cell signaling and interaction.
2	***ARG***, ***BMX***, IL-6, ***KCNJ2***, TNFSTF14, TREM1	Cell death and survival, inflammatory diseases, inflammatory response.
3	***HLA-DRB4***, PRTN3, ***SLPI***, SPHK1, TNF	Inflammatory response, immune cell trafficking, cell-to-cell signaling and interaction.

To understand the inflammatory related regulation shared by both RA and CAD, we predicted the upstream regulators of the DEGs in RA *vs*. normal controls and CAD *vs*. normal controls by IPA. The activation of three upstream regulators shared in both RA and CAD were predicted by the IPA platform: TNF, IL1β and p38 MAPK. The networks of upstream regulators and target molecules in RA and CAD are drawn in **Fig. 5D and 5E** and **Table 4**. Additionally, the shared downstream target DEGs were as follows: MMP9, EGR2, CTGF, HLA-DRB4 and RBP1 were found within both RA and CAD (**Table 4**). The top shared function of the predicted upstream regulator networks was also focused on the inflammatory response between RA and CAD (**Table 4**).

**Table 4:** The top functions of predicted upstream regulators networks in RA *vs*. normal and CAD *vs*. normal and targets molecules involved in (The molecules in italics are shared predicted upstream regulators and targets in both RA *vs.* normal and CAD *vs.* normal groups).

Groups	Predicted upstream regulators	Targets of upstream regulators	Top functions of predicted upstream regulators networks
RA *vs.* normal	IgG, ***p38*** ** ***MAPK***, TGFB1, ***TNF***, TREM1, EIF2AK2, IFNG, IFNL1, IL13, ***IL1B***, IRF3	ADA, ADM, ARG1, BIRC5, C1QB, C1QC, CCL2/MCP-1, ***CTGF***, CXCL1, EGR1, ***EGR2***, FN1, GSTA1, ***HLA-DRB4***, IFI27, IFI44L, IFIT1, IFIT3, IFITM3, ISG15, ITGB4, KCNJ2, KCNJ15, ***MMP9***, NOV, ***RBP1***, SNAI1, TNFSF11	Inflammatory response, connective tissue disorders, inflammatory disease.
CAD *vs.* normal	NF-κB (complex), ***P38*** ** ***MAPK***, ***IL1B***, ***TNF***	APOBEC3B, CAMP, CCL3, CCL3L1/CCL3L3, CHI3L1, ***CTGF***, ***EGR2***, ***HLA-DRB4***, MMP8, ***MMP9***, PTGES, ***RBP1***	Inflammatory response, hypersensitivity response, cardiovascular disease.

## Discussion

Both RA and CAD are reported to be chronic inflammatory disease, and usually occur sequentially or exist together, leading to high mortality rates [35], [36]. Sattar *et al.* suggested markers of systemic inflammation (such as interleukin (IL)-6, IL-1, and tumor necrosis factor (TNF)) have been correlated with an increased risk of cardiovascular death in patients with RA [37]. TNF-α and IL-6 have been reported to be significantly associated with the severity of subclinical atherosclerosis, independent of Framingham risk score in RA [15], [38], and IL-6 was recently also documented to be very strongly and independently associated with surrogate markers of early atherogenesis in RA [15]. Most of those studies were based on hypothesis-driven candidate genes or inflammatory factors associated or performed in RA and CAD patients. However, how inflammatory modifications were made, the gene profile and the related pathways shared within the two disorders were not fully understood [39].

**Figure 6:**
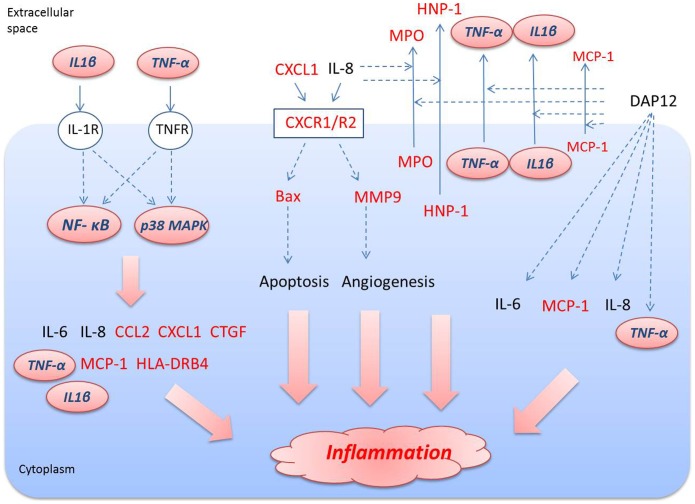
The summary view of shared pathways related with shared DEGs in RA and CAD involved in inflammation. The molecules with red color represent the shared DEGs in both RA *vs*. normal and CAD *vs*. normal; molecules with italics in pink shapes represent the shared upstream regulators in both RA vs. normal and CAD *vs*. normal.

In the present study, we found 19 of the most important DEGs shared between RA and CAD. Based on the top shared functions, 4 shared canonical pathways, 3 shared associated networks, 3 upstream regulators predicted activation and the key DEGs involved in, we draw the summary path gram involved in inflammation in RA and CAD (**Fig. 6**), which may be considered as new targets or target networks for the prevention and treatment of patients with CAD and RA simultaneously.

The IL-8 signaling pathway was found to be the most important inflammatory signaling pathway shared both in RA and CAD, in which 7 identified DEGs were found to be commonly shared: BAX, CXCL1, CXCR1, CXCR2, DEFA1, MMP9 and MPO. Significant evidence implicates IL-8 as a major mediator of inflammation and joint destruction in rheumatoid arthritis [40]. The effects of IL-8 and its related ligands are mediated via two receptors, CXCR1 and CXCR2. Several studies have reported that CXCR1/2 mediate neutrophil migration and are involved in the cascade of events leading to inflammation. In addition to modifying fundamental pathological processes, non-competitive allosteric inhibitors of CXCR1/2 may have the additional benefit of providing partial relief for pain and, hence, may be a valid therapeutic target for the treatment of RA [41], [42]. In addition, we can also find reports about the increased levels of IL-8, CXCR1 and CXCR2 in CAD patients, which is supposed to be involved in the pathogenic mechanisms in heart failure [43], [44]. In our study, we acquired similar results to other reports: CXCR1 and CXCR2 can be up-regulated in both RA and CAD patients. Many MMPs are expressed at increased levels in RA tissues and in synoviocyte cultures in response to inflammatory cytokines including MMP9 [45]. Additionally, several mechanism and clinical studies support that MMP9 is a risk factor for CAD [46], [47]. MMP9 was up-regulated in both RA and CAD patients compared to the healthy controls in our study, which can lead to an inflammatory response through the regulation of endothelial cell migration during angiogenesis [48], [49]. Bax, a key node during apoptotic mechanisms participating in inflammation, was reported to be active in several studies of the pathogenesis of both RA and CAD [50], [51]. In response to inflammatory events, activated neutrophils release myeloperoxidase (MPO), which has been reported in both RA and CAD patients as well [38], [50]. In addition, we found the induction of the defensin gene: DEFA1/HNP-1 (defensin, alpha 1) was also interpreted as being up-regulated in peripheral blood in both RA and CAD. Bovin LF, *et al.* had confirmed that there was a significantly higher expression of DEFA1/HNP-1 in RA patients than in healthy controls [51], suggesting a role for defensins in the pathogenesis of RA. However, we did not find reports about the role that DEFA plays in the pathological process of CAD. Thus, DEFA1 may be considered a novel shared drug target in both RA and CAD.

The other shared signaling pathways, antigen presentation pathway, OX40 signaling pathway and TREM1 signaling, were found to participate in inflammatory process overlap or crossover with the IL-8 signaling, as shown in **Fig. 4** **,** **Table 2**. Chemokine (C-C motif) ligand 2 (CCL2) is referred to as monocyte chemotactic protein-1 (MCP-1) and is an important risk factor through its role in recruiting circulating monocytes to sites of atherosclerotic lesions [52], [53]. In RA, MCP-1/CCL2 regulate up activation of normal T cell expression and secretion of (RANTES)/CCL5, growth-regulated oncogene α (GROα)/CXCL1 and IL-8/CXCL8, which are potent chemotactic agents constitutively produced by RA synovial fibroblasts and further up-regulated upon cytokine stimulation [54]. Both HLA-DRB4 and HLA-DQA2 belong to class II histocompatibility antigens, which are expressed in antigen presenting cells (B lymphocytes, dendritic cells, macrophages) and are used to present antigenic peptides on the cell surface to be recognized by CD4 T-cells during inflammation, reported in the RA process in many papers [55]. In particular, the HLA-DRB1 gene was reported to be not only a risk factor for RA but also for CAD disease [56]. DRB4 is linked with allelic variants of DRB1, but is otherwise omitted and reported less frequently in RA or CAD. HLA-DQA2 was also associated with rheumatoid arthritis [57], but less frequently reported in CAD [58].

TNF, IL1β and p38 MAPK were predicted to be activated as up-stream regulators shared in both RA and CAD in this study. Most of the shared DEGs were regulated by the three regulators leading to the inflammatory response in the end, such as CTGF, EGR2, MMP9, CXCL1, BAX, HLA-DRB4, CCL2/MCP-1 and MPO (**Fig. 6**). As we know, p38 MAPK, TNF and IL1β play important roles in transducing inflammation, by which several transcription factors can be directly phosphorylated and activated to bring about pro-inflammatory factors in RA, CAD and other inflammatory diseases [59], [60]. Therefore, these regulators were also considered as key molecular targets for therapeutic intervention in chronic inflammatory diseases such as RA or CAD [61], [62]. For example, the inhibitors of p38 MAPK RWJ67657 and BIRB-796 have been tested in clinical studies in humans [62]. TNF inhibitors have proven their clinical efficacy in the treatment of RA, but the targeted regulation of TNF response for therapeutic benefits remains suboptimal [63]. In some reports, treatment of CAD patients with TNF-α antagonist did not show encouraging results and was even associated with worsening of cardiac events in some instances [64]. However, some others found that using TNF-α inhibitors was associated with a decreased risk for CAD in RA [65]. Oppositely, it was reported that neither treatment with, nor response to, anti-TNF therapy could be linked to any statistically significant decrease in the risk of acute coronary syndromes [66]. Perhaps tailored anti-TNF-α therapy in relation to the TNF-α genotype of CAD patients and targeting of the cytokine gene expression via signaling pathway inhibitors may have useful clinical implications [64]. The same time, preclinical and clinical studies of IL-1β inhibition have shown efficacy in the treatment of several inflammatory disorders, suggesting that IL-1β may be a novel therapeutic target for anti-inflammatory therapy such as CAD or RA [67], [68]. For example, Anakinra (Kineret), a recombinant form of human interleukin-1 (IL-1) receptor antagonist, is approved for the treatment of RA in combination with methotrexate [68]. All of those regulator antagonists are single target drugs, only inhibiting certain pathogenetic targets to intervene in RA or CAD independently, and leading to several side effects at the same time. Thus, exploiting the combination drugs that can act on the shared multiple targets or network targets may be considered a novel therapeutic and preventive strategy for curing RA and CAD at the same time. For example, a drug that targets the p38 MAPK, TNF and IL1β at the same time or that targets the shared inflammatory molecular network may be the next topic of research.

## Limitations

Some potential limitations of the study should be considered. First, the patients participating in this study had either RA or CAD alone; patients with RA combined CAD were not included because of the complex situation of inflammation [69] and the lack of a relatively accepted inclusion criteria with integration of these two diseases. The verification of the shared inflammatory biomarker of the patients with both RA and CAD will be our future work. Additionally, the number of patients included in the study was small, partly because of funding constraints; a larger sample is needed to further verify the results of this study.

## Conclusions

In summary, the present study gave a global understanding of the shared inflammatory characteristics in both RA and CAD. IL-8 signaling, antigen presentation, OX40 signaling and TREM1 signaling based on canonical pathways and the 19 shared DEGs involved were found to be the commonly shared inflammatory characteristics of RA and CAD. In particular, p38 MAPK, TNF and IL1β were predicted to be commonly activated in RA and CAD. These shared pathways, key regulators and down-stream cytokines of inflammation may be considered to be novel targets or network targets for the treatment and prevention of RA and CAD at the same time. Further studies are needed to develop and confirm new drugs that can concurrently target the pathways of both RA and CAD or to predict when CAD will develop in RA patients in depth.

## Supporting Information

S1 File **Supporting tables.** Table S1. Differentially expressed genes of PBMCs in RA patients compared with normal control. Table S2. Differentially expressed genes of PBMCs in CAD patients compared with normal control. Table S3. The shared 79 differentially expressed genes in both RA and CAD patients compared with normal control.
(DOCX)
