# Supplementary material for: The Shared Crosstalk of Multiple Pathways Involved in the Inflammation between Rheumatoid Arthritis and Coronary Artery Disease Based on a Digital Gene Expression Profile
Source: PLoS One. 2014 Dec 16;9(12):e113659. doi: 10.1371/journal.pone.0113659 (PMC4267808; doi:10.1371/journal.pone.0113659)
Supplement: S1 File — Supporting tables. Table S1. Differentially expressed genes of PBMCs in RA patients compared with normal control. Table S2. Differentially expressed genes of PBMCs in CAD patients compared with normal control. Table S3. The shared 79 differentially expressed genes in both RA and CAD patients compared with normal control. (DOCX) [file pone.0113659.s001.docx]

**Supporting Tables:**

**Supporting Table S1. Differentially expressed genes of PBMCs in RA patients compared with normal control**

| **No.** | **Symbol** | **log2 Ratio(RA/normal)** | ***P*-value** | **FDR** |
| --- | --- | --- | --- | --- |
| 1 | ***MIR508*** | 5.64 | 1.36E-09 | 9.27E-09 |
| 2 | ***T*** | 5.17 | 3.78E-07 | 2.07E-06 |
| 3 | ***KIR2DS5*** | 5.09 | 7.65E-07 | 4.05E-06 |
| 4 | ***BEX2*** | 5.04 | 1.55E-06 | 7.88E-06 |
| 5 | ***CTGF*** | 4.95 | 3.12E-06 | 1.53E-05 |
| 6 | ***GBP1P1*** | 4.95 | 3.12E-06 | 1.53E-05 |
| 7 | ***ASPRV1*** | 4.86 | 6.31E-06 | 2.98E-05 |
| 8 | ***SNCB*** | 4.81 | 1.28E-05 | 5.77E-05 |
| 9 | ***RNF17*** | 4.81 | 1.28E-05 | 5.77E-05 |
| 10 | ***FN1*** | 4.71 | 1.72E-05 | 7.64E-05 |
| 11 | ***ZKSCAN4*** | 4.70 | 2.58E-05 | 1.11E-04 |
| 12 | ***METTL7B*** | 4.58 | 5.21E-05 | 2.15E-04 |
| 13 | ***MET*** | 4.46 | 0.000105 | 4.16E-04 |
| 14 | ***LOC100132215*** | 4.39 | 0.000213 | 7.99E-04 |
| 15 | ***TBX15*** | 4.39 | 0.000213 | 7.99E-04 |
| 16 | ***LOC647012*** | 4.39 | 0.000213 | 7.99E-04 |
| 17 | ***BMX*** | 4.05 | 7.58E-05 | 3.06E-04 |
| 18 | ***FCGR3B*** | 3.92 | 1.79E-06 | 9.06E-06 |
| 19 | ***RNF182*** | 3.46 | 7.60E-05 | 3.07E-04 |
| 20 | ***C4BPA*** | 3.32 | 1.38E-11 | 1.27E-10 |
| 21 | ***SLC26A8*** | 3.02 | 1.79E-06 | 9.06E-06 |
| 22 | ***C1QC*** | 3.00 | 1.35E-13 | 1.95E-12 |
| 23 | ***BTNL3*** | 3.00 | 9.97E-06 | 4.58E-05 |
| 24 | ***HBZ*** | 2.89 | 5.05E-05 | 2.09E-04 |
| 25 | ***PRDM16*** | 2.89 | 5.05E-05 | 2.09E-04 |
| 26 | ***IFI27*** | 2.76 | 0.00 | 0.00 |
| 27 | ***AEBP1*** | 2.64 | 4.62E-05 | 1.92E-04 |
| 28 | ***STOX1*** | 2.58 | 1.93E-05 | 8.50E-05 |
| 29 | ***KCNMA1*** | 2.57 | 5.45E-07 | 2.93E-06 |
| 30 | ***OTOF*** | 2.53 | 9.50E-14 | 1.38E-12 |
| 31 | ***EGR2*** | 2.50 | 0.00 | 0.00 |
| 32 | ***LOC642236*** | 2.43 | 1.13E-05 | 5.13E-05 |
| 33 | ***CD177*** | 2.40 | 0.00 | 0.00 |
| 34 | ***CCNB2*** | 2.39 | 1.26E-13 | 1.81E-12 |
| 35 | ***C7orf25*** | 2.37 | 8.04E-06 | 3.75E-05 |
| 36 | ***C1QB*** | 2.36 | 0.00 | 0.00 |
| 37 | ***NOV*** | 2.35 | 2.98E-07 | 1.65E-06 |
| 38 | ***NOP16*** | 2.33 | 4.74E-08 | 2.83E-07 |
| 39 | ***SIGLEC1*** | 2.32 | 5.68E-13 | 7.58E-12 |
| 40 | ***CNTNAP3*** | 2.27 | 2.12E-05 | 9.28E-05 |
| 41 | ***CA4*** | 2.26 | 1.68E-10 | 1.26E-09 |
| 42 | ***ARG1*** | 2.22 | 3.89E-14 | 5.79E-13 |
| 43 | ***PSMD4*** | 2.20 | 0.000171 | 6.53E-04 |
| 44 | ***SLPI*** | 2.18 | 2.64E-06 | 1.30E-05 |
| 45 | ***SPESP1*** | 2.16 | 3.20E-06 | 1.57E-05 |
| 46 | ***IGFL2*** | 2.16 | 3.71E-05 | 1.56E-04 |
| 47 | ***MME*** | 2.11 | 0.00 | 0.00 |
| 48 | ***ADA*** | 2.08 | 2.31E-12 | 2.76E-11 |
| 49 | ***CCL2*** | 2.07 | 0.00 | 0.00 |
| 50 | ***BPI*** | 2.06 | 8.22E-06 | 3.83E-05 |
| 51 | ***HSPA6*** | 2.06 | 0.00 | 0.00 |
| 52 | ***CNTNAP3B*** | 2.02 | 3.27E-06 | 1.60E-05 |
| 53 | ***PLIN4*** | 2.02 | 3.32E-10 | 2.40E-09 |
| 54 | ***CCR9*** | 1.99 | 9.50E-14 | 1.39E-12 |
| 55 | ***DLGAP5*** | 1.96 | 3.29E-08 | 1.99E-07 |
| 56 | ***CXCR1*** | 1.96 | 0.00 | 0.00 |
| 57 | ***HBG1*** | 1.95 | 0.00 | 0.00 |
| 58 | ***IFIT3*** | 1.92 | 0.00 | 0.00 |
| 59 | ***UHRF1*** | 1.92 | 8.97E-10 | 6.20E-09 |
| 60 | ***PGLYRP1*** | 1.91 | 0.00 | 0.00 |
| 61 | ***MMP9*** | 1.90 | 2.72E-13 | 3.81E-12 |
| 62 | ***TPX2*** | 1.90 | 0.00 | 0.00 |
| 63 | ***FOLR3*** | 1.85 | 0.00 | 0.00 |
| 64 | ***ALPL*** | 1.85 | 3.55E-15 | 5.46E-14 |
| 65 | ***DEFA3*** | 1.85 | 1.92E-12 | 2.35E-11 |
| 66 | ***KCNJ2*** | 1.84 | 2.24E-13 | 3.17E-12 |
| 67 | ***EGR1*** | 1.84 | 1.36E-11 | 1.25E-10 |
| 68 | ***RPL37A*** | 1.83 | 0.00 | 0.00 |
| 69 | ***CXCL1*** | 1.82 | 0.00 | 0.00 |
| 70 | ***IFIT1*** | 1.81 | 4.87E-12 | 5.11E-11 |
| 71 | ***ANKRD22*** | 1.78 | 0.00 | 0.00 |
| 72 | ***CYP4F3*** | 1.77 | 0.00 | 0.00 |
| 73 | ***RNASE1*** | 1.75 | 3.79E-09 | 2.50E-08 |
| 74 | ***HLA-DRB4*** | 1.71 | 0.00 | 0.00 |
| 75 | ***KRT23*** | 1.71 | 0.00 | 0.00 |
| 76 | ***LTF*** | 1.71 | 0.00 | 0.00 |
| 77 | ***ASPM*** | 1.71 | 0.00 | 0.00 |
| 78 | ***C17orf56*** | 1.70 | 0.00 | 0.00 |
| 79 | ***C5orf4*** | 1.67 | 0.00 | 0.00 |
| 80 | ***KCNJ15*** | 1.66 | 5.49E-13 | 7.36E-12 |
| 81 | ***AHSP*** | 1.65 | 0.00 | 0.00 |
| 82 | ***FHDC1*** | 1.63 | 5.52E-09 | 3.61E-08 |
| 83 | ***SIGLEC5*** | 1.63 | 6.99E-08 | 4.11E-07 |
| 84 | ***MCM10*** | 1.63 | 2.06E-06 | 1.04E-05 |
| 85 | ***CDT1*** | 1.62 | 0.00 | 0.00 |
| 86 | ***C8orf38*** | 1.62 | 1.17E-13 | 1.69E-12 |
| 87 | ***ADM*** | 1.61 | 0.00 | 0.00 |
| 88 | ***C19orf71*** | 1.60 | 9.72E-05 | 0.000386 |
| 89 | ***BIRC5*** | 1.60 | 4.75E-14 | 7.07E-13 |
| 90 | ***BATF2*** | 1.59 | 5.15E-14 | 7.63E-13 |
| 91 | ***MYL4*** | 1.59 | 0.00 | 0.00 |
| 92 | ***RPGRIP1*** | 1.58 | 4.82E-06 | 2.31E-05 |
| 93 | ***MND1*** | 1.58 | 1.02E-06 | 5.30E-06 |
| 94 | ***BAMBI*** | 1.58 | 4.82E-06 | 2.31E-05 |
| 95 | ***PBK*** | 1.58 | 5.29E-08 | 3.15E-07 |
| 96 | ***TIMD4*** | 1.56 | 7.33E-06 | 3.44E-05 |
| 97 | ***CEP55*** | 1.55 | 2.14E-07 | 1.20E-06 |
| 98 | ***FAM154B*** | 1.55 | 0.00 | 0.00 |
| 99 | ***IFI44L*** | 1.54 | 0.00 | 0.00 |
| 100 | ***HARBI1*** | 1.54 | 2.29E-05 | 9.99E-05 |
| 101 | ***MACROD2*** | 1.54 | 5.15E-14 | 7.64E-13 |
| 102 | ***IFITM3*** | 1.53 | 0.00 | 0.00 |
| 103 | ***ISG15*** | 1.52 | 0.00 | 0.00 |
| 104 | ***KIAA0101*** | 1.52 | 0.00 | 0.00 |
| 105 | ***MMP25*** | 1.52 | 5.90E-12 | 6.03E-11 |
| 106 | ***BTNL8*** | 1.51 | 4.61E-06 | 2.22E-05 |
| 107 | ***KREMEN1*** | 1.50 | 1.02E-11 | 9.73E-11 |
| 108 | ***FCGR1A*** | 1.50 | 0.00 | 0.00 |
| 110 | ***SKA1*** | 1.50 | 1.96E-05 | 8.64E-05 |
| 111 | ***PHOSPHO1*** | 1.48 | 7.39E-14 | 1.08E-12 |
| 112 | ***USP18*** | 1.48 | 2.26E-13 | 3.18E-12 |
| 113 | ***FCER1G*** | 1.48 | 8.50E-13 | 1.11E-11 |
| 114 | ***CMTM2*** | 1.47 | 0.00 | 0.00 |
| 115 | ***S100P*** | 1.47 | 3.13E-12 | 3.55E-11 |
| 116 | ***RSAD2*** | 1.47 | 2.25E-13 | 3.17E-12 |
| 117 | ***TOP2A*** | 1.46 | 0.00 | 0.00 |
| 118 | ***PRKCDBP*** | 1.45 | 5.32E-07 | 2.86E-06 |
| 119 | ***TYMS*** | 1.43 | 5.81E-13 | 7.75E-12 |
| 120 | ***IL1R2*** | 1.43 | 0.00 | 0.00 |
| 121 | ***CDC6*** | 1.42 | 2.57E-08 | 1.57E-07 |
| 122 | ***KIF4A*** | 1.42 | 3.28E-07 | 1.80E-06 |
| 123 | ***LRG1*** | 1.42 | 6.96E-13 | 9.19E-12 |
| 124 | ***CXCR2*** | 1.41 | 6.81E-12 | 6.80E-11 |
| 125 | ***WNT16*** | 1.40 | 2.73E-08 | 1.67E-07 |
| 126 | ***UNC13B*** | 1.40 | 3.04E-06 | 1.49E-05 |
| 127 | ***HBD*** | 1.40 | 1.79E-13 | 2.55E-12 |
| 128 | ***BAX*** | 1.39 | 2.01E-13 | 2.86E-12 |
| 129 | ***ANXA3*** | 1.37 | 1.41E-13 | 2.03E-12 |
| 130 | ***SIGLEC14*** | 1.36 | 0.00 | 0.00 |
| 131 | ***SOCS3*** | 1.36 | 2.72E-12 | 3.16E-11 |
| 132 | ***CES1*** | 1.35 | 0.00 | 0.00 |
| 133 | ***RPH3A*** | 1.35 | 1.03E-13 | 1.50E-12 |
| 134 | ***CDKN3*** | 1.34 | 0.00 | 0.00 |
| 135 | ***CCNB1*** | 1.34 | 8.34E-10 | 5.78E-09 |
| 136 | ***S100A9*** | 1.34 | 1.45E-10 | 1.10E-09 |
| 137 | ***C1QA*** | 1.33 | 2.11E-13 | 2.99E-12 |
| 138 | ***OIP5*** | 1.33 | 9.17E-07 | 4.80E-06 |
| 139 | ***DIRAS1*** | 1.32 | 6.47E-07 | 3.45E-06 |
| 140 | ***MOSC1*** | 1.31 | 1.00E-13 | 1.46E-12 |
| 141 | ***HLA-DQA2*** | 1.30 | 3.75E-12 | 4.12E-11 |
| 142 | ***PLK1*** | 1.30 | 0.00 | 0.00 |
| 143 | ***KIR2DL4*** | 1.30 | 3.02E-14 | 4.52E-13 |
| 144 | ***IFI44*** | 1.30 | 1.87E-11 | 1.64E-10 |
| 145 | ***LIN7A*** | 1.30 | 0.00 | 0.00 |
| 146 | ***C2*** | 1.30 | 3.80E-13 | 5.22E-12 |
| 147 | ***CMPK2*** | 1.29 | 1.14E-12 | 1.45E-11 |
| 148 | ***SERINC2*** | 1.29 | 1.26E-11 | 1.17E-10 |
| 149 | ***BUB1B*** | 1.29 | 2.48E-12 | 2.93E-11 |
| 150 | ***PI4KA*** | 1.28 | 0.00 | 0.00 |
| 151 | ***LETM2*** | 1.28 | 5.10E-05 | 0.000211 |
| 152 | ***C5orf32*** | 1.28 | 2.94E-13 | 4.09E-12 |
| 153 | ***CPNE9*** | 1.28 | 2.05E-05 | 9.00E-05 |
| 154 | ***KIF11*** | 1.27 | 0.00 | 0.00 |
| 155 | ***MGAM*** | 1.27 | 3.53E-13 | 4.86E-12 |
| 156 | ***PPIL6*** | 1.27 | 0.00 | 0.00 |
| 157 | ***SELENBP1*** | 1.26 | 0.00 | 0.00 |
| 158 | ***GINS2*** | 1.26 | 6.63E-07 | 3.53E-06 |
| 159 | ***CHI3L1*** | 1.26 | 4.44E-11 | 3.52E-10 |
| 160 | ***ZNF202*** | 1.26 | 4.44E-10 | 3.17E-09 |
| 161 | ***CDC45*** | 1.26 | 6.37E-09 | 4.14E-08 |
| 162 | ***TNFAIP6*** | 1.25 | 2.12E-13 | 3.01E-12 |
| 163 | ***TNFRSF10C*** | 1.25 | 1.85E-13 | 2.64E-12 |
| 164 | ***HMMR*** | 1.25 | 3.10E-11 | 2.52E-10 |
| 165 | ***INSL3*** | 1.24 | 0.00 | 0.00 |
| 166 | ***CA1*** | 1.24 | 1.23E-13 | 1.77E-12 |
| 167 | ***UBB*** | 1.24 | 0.00 | 0.00 |
| 168 | ***MX1*** | 1.24 | 0.00 | 0.00 |
| 169 | ***ACAA1*** | 1.24 | 0.00 | 0.00 |
| 170 | ***MPO*** | 1.23 | 0.00 | 0.00 |
| 171 | ***FFAR2*** | 1.23 | 2.53E-12 | 2.98E-11 |
| 172 | ***ESPL1*** | 1.23 | 8.24E-06 | 3.84E-05 |
| 173 | ***LRRC4*** | 1.23 | 0.00 | 0.00 |
| 174 | ***PRUNE2*** | 1.23 | 2.04E-05 | 8.96E-05 |
| 175 | ***NCRNA00256A*** | 1.22 | 4.49E-08 | 2.68E-07 |
| 176 | ***DEFA4*** | 1.22 | 2.15E-10 | 1.58E-09 |
| 177 | ***DHRS9*** | 1.21 | 0.00 | 0.00 |
| 178 | ***FAM177B*** | 1.21 | 4.76E-06 | 2.29E-05 |
| 179 | ***CCNA2*** | 1.21 | 0.00 | 0.00 |
| 180 | ***ANO5*** | 1.21 | 1.29E-07 | 7.41E-07 |
| 181 | ***ABTB2*** | 1.21 | 8.13E-06 | 3.79E-05 |
| 182 | ***MKI67*** | 1.21 | 0.00 | 0.00 |
| 183 | ***SDC3*** | 1.20 | 1.48E-10 | 1.11E-09 |
| 184 | ***MMP8*** | 1.20 | 4.47E-07 | 2.42E-06 |
| 185 | ***ETV7*** | 1.20 | 3.39E-12 | 3.79E-11 |
| 186 | ***RBP1*** | -7.05 | 3.80E-24 | 6.83E-23 |
| 187 | ***EFCAB4B*** | -4.75 | 1.82E-05 | 8.07E-05 |
| 188 | ***IGLL3P*** | -4.35 | 1.02E-20 | 1.74E-19 |
| 189 | ***ALS2CR11*** | -3.42 | 0.000146 | 5.64E-04 |
| 190 | ***TNFSF11*** | -3.00 | 8.27E-07 | 4.36E-06 |
| 191 | ***DSCAML1*** | -2.68 | 2.48E-05 | 1.08E-04 |
| 192 | ***ITGB4*** | -2.48 | 1.21E-07 | 6.99E-07 |
| 193 | ***NRARP*** | -2.02 | 5.46E-05 | 2.24E-04 |
| 194 | ***CDRT15L2*** | -1.70 | 2.00E-06 | 1.01E-05 |
| 195 | ***SNAI1*** | -1.67 | 2.05E-11 | 1.77E-10 |
| 196 | ***GSTA1*** | -1.51 | 5.64E-05 | 2.31E-04 |
| 197 | ***SNX7*** | -1.49 | 3.32E-05 | 1.41E-04 |
| 198 | ***DAAM1*** | -1.49 | 1.55E-08 | 9.71E-08 |
| 199 | ***CACNG6*** | -1.44 | 3.79E-05 | 1.60E-04 |
| 200 | ***KRT1*** | -1.42 | 3.11E-06 | 1.53E-05 |
| 201 | ***MTRNR2L1*** | -1.34 | 7.03E-11 | 5.47E-10 |
| 202 | ***CYP1A2*** | -1.33 | 2.33E-05 | 1.02E-04 |
| 203 | ***THEM5*** | -1.31 | 0.000251 | 9.30 E-04 |
| 204 | ***YEATS2*** | -1.30 | 8.56E-07 | 4.50E-06 |
| 205 | ***LOC100507266*** | -1.27 | 1.55E-07 | 8.83E-07 |
| 206 | ***DDX19A*** | -1.26 | 8.05E-05 | 3.24E-04 |
| 207 | ***TNNC2*** | -1.26 | 4.94E-07 | 2.67E-06 |
| 208 | ***MT2A*** | -1.25 | 0.00 | 0.00 |
| 209 | ***AREG*** | -1.24 | 6.56E-10 | 4.61E-09 |
| 210 | ***TECPR1*** | -1.24 | 9.11E-15 | 1.38E-13 |
| 211 | ***HSD17B3*** | -1.24 | 2.61E-06 | 1.29E-05 |
| 212 | ***HGD*** | -1.22 | 2.80E-29 | 5.28E-28 |
| 213 | ***CXXC1P1*** | -1.22 | 1.32E-14 | 1.99E-13 |

**Supporting Table S2. Differentially expressed genes of PBMCs in CAD patients compared with normal control**

| **No.** | **Symbol** | **log2 Ratio(CAD/normal)** | ***P*-value** | **FDR** |
| --- | --- | --- | --- | --- |
| 1 | ***MIR508*** | 5.36 | 5.52E-08 | 4.19E-07 |
| 2 | ***ZKSCAN4*** | 5.00 | 1.79E-06 | 1.10E-05 |
| 3 | ***BEX2*** | 5.00 | 1.79E-06 | 1.10E-05 |
| 4 | ***CTGF*** | 5.00 | 1.79E-06 | 1.10E-05 |
| 5 | ***CLLU1OS*** | 4.86 | 7.22E-06 | 4.02E-05 |
| 6 | ***ASPRV1*** | 4.75 | 1.45E-05 | 7.67E-05 |
| 7 | ***LOC647012*** | 4.64 | 2.91E-05 | 1.45E-04 |
| 8 | ***CCL3*** | 4.64 | 2.91E-05 | 1.45E-04 |
| 9 | ***PI15*** | 4.58 | 5.83E-05 | 2.77E-04 |
| 10 | ***C8orf85*** | 4.46 | 1.17E-04 | 5.24E-04 |
| 11 | ***LOC283392*** | 4.32 | 2.35E-04 | 9.91E-04 |
| 12 | ***LOC100129726*** | 4.32 | 2.35E-04 | 9.91E-04 |
| 13 | ***KIR2DS5*** | 4.32 | 2.35E-04 | 9.90E-04 |
| 14 | ***FCGR3B*** | 3.80 | 1.66E-06 | 1.02E-05 |
| 15 | ***LOC100129345*** | 3.77 | 7.42E-05 | 3.46E-04 |
| 16 | ***BMX*** | 3.49 | 7.43E-05 | 3.47E-04 |
| 17 | ***KRT18*** | 3.38 | 4.65E-05 | 2.25E-04 |
| 18 | ***RNF182*** | 3.07 | 8.84E-05 | 4.06E-04 |
| 19 | ***HIST1H4L*** | 3.07 | 8.84E-05 | 4.06E-04 |
| 20 | ***CCL3L1*** | 2.90 | 2.25E-08 | 1.79E-07 |
| 21 | ***RIMBP2*** | 2.81 | 5.22E-05 | 2.51E-04 |
| 22 | ***SLC26A8*** | 2.78 | 1.66E-06 | 1.02E-05 |
| 23 | ***MMP8*** | 2.65 | 4.86E-14 | 1.19E-12 |
| 24 | ***C7orf25*** | 2.64 | 5.39E-06 | 3.07E-05 |
| 25 | ***LUM*** | 2.61 | 1.80E-05 | 9.36E-05 |
| 26 | ***EGR2*** | 2.60 | 0.00 | 0.00 |
| 27 | ***LTF*** | 2.60 | 0.00 | 0.00 |
| 28 | ***MEG3*** | 2.49 | 5.73E-06 | 3.24E-05 |
| 29 | ***DEFA3*** | 2.47 | 2.93E-12 | 5.30E-11 |
| 30 | ***NOP16*** | 2.46 | 4.21E-08 | 3.25E-07 |
| 31 | ***CNTNAP3*** | 2.46 | 1.04E-05 | 5.67E-05 |
| 32 | ***DAZL*** | 2.46 | 1.34E-04 | 5.91E-04 |
| 33 | ***NOV*** | 2.41 | 2.71E-07 | 1.87E-06 |
| 34 | ***SLPI*** | 2.37 | 1.69E-06 | 1.04E-05 |
| 35 | ***CHI3L1*** | 2.35 | 1.73E-14 | 4.35E-13 |
| 36 | ***MME*** | 2.33 | 0.00 | 0.00 |
| 37 | ***FSIP1*** | 2.29 | 8.10E-05 | 3.75E-04 |
| 38 | ***COL17A1*** | 2.28 | 1.38E-05 | 7.33E-05 |
| 39 | ***MMP9*** | 2.26 | 4.49E-13 | 9.95E-12 |
| 40 | ***ADA*** | 2.23 | 1.90E-12 | 3.62E-11 |
| 41 | ***GPC1*** | 2.23 | 1.25E-04 | 5.57E-04 |
| 42 | ***CA4*** | 2.23 | 1.40E-10 | 1.45E-09 |
| 43 | ***CEACAM8*** | 2.21 | 2.45E-13 | 5.67E-12 |
| 44 | ***PGLYRP1*** | 2.17 | 2.22E-16 | 5.78E-15 |
| 45 | ***ARG1*** | 2.16 | 6.68E-14 | 1.62E-12 |
| 46 | ***C4BPA*** | 2.15 | 1.13E-11 | 1.61E-10 |
| 47 | ***CD177*** | 2.10 | 0.00 | 0.00 |
| 48 | ***KRT23*** | 2.10 | 0.00 | 0.00 |
| 49 | ***OLFM4*** | 2.07 | 0.00 | 0.00 |
| 50 | ***KAZN*** | 2.06 | 2.00E-07 | 1.41E-06 |
| 51 | ***ANXA3*** | 2.04 | 2.22E-16 | 5.77E-15 |
| 52 | ***BTNL3*** | 2.03 | 1.87E-04 | 8.06E-04 |
| 53 | ***PTGES*** | 2.02 | 4.01E-05 | 1.96E-04 |
| 54 | ***DEFA4*** | 2.00 | 0.00 | 0.00 |
| 55 | ***HLA-DRB4*** | 1.99 | 0.00 | 0.00 |
| 56 | ***BAX*** | 1.98 | 3.07E-13 | 7.01E-12 |
| 57 | ***KCNJ2*** | 1.98 | 3.11E-13 | 7.09E-12 |
| 58 | ***RGL3*** | 1.94 | 8.76E-06 | 4.80E-05 |
| 59 | ***GPR162*** | 1.88 | 1.54E-06 | 9.49E-06 |
| 60 | ***OLR1*** | 1.83 | 3.17E-13 | 7.22E-12 |
| 61 | ***OTOF*** | 1.82 | 1.26E-13 | 2.99E-12 |
| 62 | ***DOCK1*** | 1.78 | 5.83E-06 | 3.29E-05 |
| 63 | ***BPI*** | 1.77 | 2.30E-04 | 9.74E-04 |
| 64 | ***CNTNAP3B*** | 1.73 | 1.31E-04 | 5.81E-04 |
| 65 | ***LRG1*** | 1.72 | 0.00 | 0.00 |
| 66 | ***LOC100130298*** | 1.71 | 1.01E-06 | 6.36E-06 |
| 67 | ***CRISP3*** | 1.69 | 0.00 | 0.00 |
| 68 | ***C5orf4*** | 1.66 | 0.00 | 0.00 |
| 69 | ***CAMP*** | 1.64 | 4.77E-14 | 1.17E-12 |
| 70 | ***CEACAM6*** | 1.63 | 7.90E-08 | 5.89E-07 |
| 71 | ***ZNF230*** | 1.63 | 1.61E-04 | 6.99E-04 |
| 72 | ***CXCR1*** | 1.62 | 4.06E-14 | 1.00E-12 |
| 73 | ***ALPL*** | 1.61 | 1.01E-13 | 2.42E-12 |
| 74 | ***C8orf38*** | 1.61 | 1.95E-13 | 4.55E-12 |
| 75 | ***CMTM2*** | 1.60 | 0.00 | 0.00 |
| 76 | ***PLIN4*** | 1.57 | 1.26E-06 | 7.89E-06 |
| 77 | ***GPR97*** | 1.56 | 7.30E-13 | 1.54E-11 |
| 78 | ***ID3*** | 1.55 | 3.02E-13 | 6.91E-12 |
| 79 | ***NECAB2*** | 1.55 | 3.80E-06 | 2.22E-05 |
| 80 | ***CYP4F3*** | 1.54 | 0.00 | 0.00 |
| 81 | ***C5orf54*** | 1.54 | 2.13E-07 | 1.49E-06 |
| 82 | ***RPL37A*** | 1.54 | 0.00 | 0.00 |
| 83 | ***KCNJ15*** | 1.53 | 7.11E-13 | 1.51E-11 |
| 84 | ***ADAMTS1*** | 1.52 | 3.64E-14 | 9.00E-13 |
| 85 | ***SOBP*** | 1.52 | 6.07E-05 | 2.88E-04 |
| 86 | ***HLA-DQA2*** | 1.51 | 5.06E-12 | 8.19E-11 |
| 87 | ***ARFGAP1*** | 1.51 | 1.87E-14 | 4.67E-13 |
| 88 | ***TNFSF14*** | 1.50 | 0.00 | 0.00 |
| 89 | ***CXCL1*** | 1.47 | 0.00 | 0.00 |
| 90 | ***EGR1*** | 1.45 | 1.65E-11 | 2.21E-10 |
| 91 | ***C17orf56*** | 1.44 | 5.13E-14 | 1.25E-12 |
| 92 | ***MPO*** | 1.43 | 0.00 | 0.00 |
| 93 | ***CCL2*** | 1.43 | 0.00 | 0.00 |
| 94 | ***CN5H6.4*** | 1.43 | 1.01E-04 | 4.59E-04 |
| 95 | ***BTNL8*** | 1.42 | 2.09E-05 | 1.08E-04 |
| 96 | ***PHLDA3*** | 1.39 | 5.30E-08 | 4.03E-07 |
| 97 | ***CTSG*** | 1.39 | 1.97E-10 | 1.98E-09 |
| 98 | ***HES2*** | 1.39 | 6.25E-05 | 2.95E-04 |
| 99 | ***HBD*** | 1.38 | 3.00E-13 | 6.87E-12 |
| 100 | ***PARS2*** | 1.38 | 2.65E-07 | 1.84E-06 |
| 101 | ***HRH4*** | 1.36 | 4.79E-08 | 3.66E-07 |
| 102 | ***HSD17B7P2*** | 1.35 | 3.82E-05 | 1.87E-04 |
| 103 | ***UHRF1*** | 1.33 | 7.77E-05 | 3.61E-04 |
| 104 | ***CEBPE*** | 1.32 | 2.49E-14 | 6.18E-13 |
| 105 | ***CXCR2*** | 1.32 | 7.69E-12 | 1.15E-10 |
| 106 | ***MIR548O*** | 1.31 | 2.91E-07 | 1.99E-06 |
| 107 | ***TCN1*** | 1.31 | 4.22E-15 | 1.07E-13 |
| 108 | ***TCL1A*** | 1.31 | 8.19E-12 | 1.22E-10 |
| 109 | ***IMP4*** | 1.30 | 1.80E-08 | 1.45E-07 |
| 110 | ***VNN3*** | 1.30 | 2.77E-13 | 6.39E-12 |
| 110 | ***FCGR3A*** | 1.29 | 4.67E-09 | 4.01E-08 |
| 111 | ***AHSP*** | 1.27 | 5.02E-14 | 1.23E-12 |
| 112 | ***ELANE*** | 1.25 | 3.87E-13 | 8.69E-12 |
| 113 | ***MGAM*** | 1.25 | 5.68E-13 | 1.23E-11 |
| 114 | ***CEACAM1*** | 1.25 | 3.58E-13 | 8.06E-12 |
| 115 | ***TNFRSF10C*** | 1.22 | 4.70E-13 | 1.03E-11 |
| 116 | ***FAM177B*** | 1.22 | 3.51E-06 | 2.06E-05 |
| 117 | ***SLC22A16*** | 1.22 | 1.00E-05 | 5.45E-05 |
| 118 | ***PI4KA*** | 1.22 | 3.32E-13 | 7.53E-12 |
| 119 | ***HLA-L*** | 1.21 | 7.57E-13 | 1.59E-11 |
| 120 | ***HSPA6*** | 1.21 | 0.00 | 0.00 |
| 121 | ***LOC387647*** | 1.21 | 4.52E-10 | 4.35E-09 |
| 122 | ***PHOSPHO1*** | 1.21 | 2.45E-13 | 5.66E-12 |
| 123 | ***ZNF160*** | 1.21 | 5.79E-05 | 2.75E-04 |
| 124 | ***FCER1G*** | 1.21 | 1.43E-12 | 2.80E-11 |
| 125 | ***CA6*** | 1.20 | 1.55E-07 | 1.11E-06 |
| 126 | ***C1QB*** | 1.20 | 8.32E-13 | 1.73E-11 |
| 127 | ***LARGE*** | 1.20 | 4.38E-07 | 2.93E-06 |
| 128 | ***CCNB2*** | 1.20 | 7.05E-06 | 3.94E-05 |
| 129 | ***NETO1*** | 1.20 | 1.17E-04 | 5.25E-04 |
| 130 | ***CYP4F12*** | 1.20 | 1.66E-04 | 7.19E-04 |
| 131 | ***CCDC121*** | 1.20 | 4.11E-06 | 2.38E-05 |
| 132 | ***MCOLN3*** | 1.20 | 8.65E-07 | 5.53E-06 |
| 133 | ***RNASE3*** | 1.20 | 1.76E-13 | 4.13E-12 |
| 134 | ***HLA-DOB*** | 1.20 | 2.11E-14 | 5.25E-13 |
| 135 | ***IL1R2*** | 1.20 | 8.62E-14 | 2.08E-12 |
| 136 | ***HIST1H2BK*** | 1.20 | 1.40E-05 | 7.42E-05 |
| 137 | ***RBP1*** | -7.06 | 2.11E-24 | 6.00E-23 |
| 138 | ***IGLL3P*** | -5.576 | 3.28E-23 | 9.19E-22 |
| 139 | ***LOC100505875*** | -2.036 | 7.36E-15 | 1.85E-13 |
| 140 | ***APOBEC3B*** | -1.97 | 3.05E-07 | 2.08E-06 |
| 141 | ***SNORA72*** | -1.64 | 1.82E-06 | 1.11E-05 |
| 142 | ***C15orf54*** | -1.55 | 1.13E-06 | 7.08E-06 |
| 143 | ***MT2A*** | -1.53 | 0.00 | 0.00 |
| 144 | ***NR4A2*** | -1.41 | 8.09E-57 | 2.62E-55 |
| 145 | ***CELP*** | -1.39 | 1.84E-04 | 7.93E-04 |
| 146 | ***MTRNR2L1*** | -1.39 | 1.87E-11 | 2.44E-10 |
| 147 | ***KRT73*** | -1.35 | 3.40E-09 | 2.97E-08 |
| 148 | ***SNAI1*** | -1.26 | 4.95E-08 | 3.78E-07 |
| 149 | ***TAF7L*** | -1.26 | 4.06E-09 | 3.51E-08 |
| 150 | ***TREML3*** | -1.24 | 2.64E-05 | 1.33E-04 |
| 151 | ***COL5A3*** | -1.22 | 1.24E-13 | 2.94E-12 |
| 152 | ***RETN*** | -1.20 | 1.12E-54 | 3.59E-53 |

**Supporting Table S3. The shared 79 differentially expressed genes in both RA and CAD patients compared with normal control**

| **No.** | **Gene Name** | **log2 Ratio(RA/normal)** | |
| --- | --- | --- | --- |
|  |  | **RA *vs.* normal** | **CAD *vs.* normal** |
| 1 | ***MIR508*** | 5.64 | 5.36 |
| 2 | ***KIR2DS5*** | 5.09 | 4.32 |
| 3 | ***BEX2*** | 5.04 | 5.00 |
| 4 | ***CTGF*** | 4.95 | 5.00 |
| 5 | ***ASPRV1*** | 4.86 | 4.75 |
| 6 | ***ZKSCAN4*** | 4.70 | 5.00 |
| 7 | ***LOC647012*** | 4.39 | 4.64 |
| 8 | ***BMX*** | 4.05 | 3.49 |
| 9 | ***FCGR3B*** | 3.92 | 3.80 |
| 10 | ***RNF182*** | 3.46 | 3.07 |
| 11 | ***C4BPA*** | 3.32 | 2.15 |
| 12 | ***SLC26A8*** | 3.02 | 2.78 |
| 13 | ***BTNL3*** | 3.00 | 2.03 |
| 14 | ***OTOF*** | 2.53 | 1.82 |
| 15 | ***EGR2*** | 2.50 | 2.60 |
| 16 | ***CD177*** | 2.40 | 2.10 |
| 17 | ***C7orf25*** | 2.37 | 2.64 |
| 18 | ***C1QB*** | 2.36 | 1.20 |
| 19 | ***NOV*** | 2.35 | 2.41 |
| 20 | ***NOP16*** | 2.33 | 2.46 |
| 21 | ***CNTNAP3*** | 2.27 | 2.46 |
| 22 | ***CA4*** | 2.26 | 2.23 |
| 23 | ***ARG1*** | 2.22 | 2.16 |
| 24 | ***SLPI*** | 2.18 | 2.37 |
| 25 | ***MME*** | 2.11 | 2.33 |
| 26 | ***ADA*** | 2.08 | 2.23 |
| 27 | ***CCL2*** | 2.07 | 1.43 |
| 28 | ***BPI*** | 2.06 | 1.77 |
| 29 | ***HSPA6*** | 2.06 | 1.21 |
| 30 | ***CNTNAP3B*** | 2.02 | 1.73 |
| 31 | ***PLIN4*** | 2.02 | 1.57 |
| 32 | ***CXCR1*** | 1.96 | 1.62 |
| 33 | ***UHRF1*** | 1.92 | 1.33 |
| 34 | ***PGLYRP1*** | 1.91 | 2.17 |
| 35 | ***MMP9*** | 1.90 | 2.26 |
| 36 | ***ALPL*** | 1.85 | 1.61 |
| 37 | ***DEFA3*** | 1.85 | 2.47 |
| 38 | ***KCNJ2*** | 1.85 | 1.98 |
| 39 | ***EGR1*** | 1.84 | 1.45 |
| 40 | ***RPL37A*** | 1.83 | 1.54 |
| 41 | ***CXCL1*** | 1.82 | 1.47 |
| 42 | ***CYP4F3*** | 1.77 | 1.54 |
| 43 | ***HLA-DRB4*** | 1.71 | 1.99 |
| 44 | ***KRT23*** | 1.71 | 2.10 |
| 45 | ***LTF*** | 1.71 | 2.60 |
| 46 | ***C17orf56*** | 1.70 | 1.44 |
| 47 | ***C5orf4*** | 1.67 | 1.66 |
| 48 | ***KCNJ15*** | 1.66 | 1.53 |
| 49 | ***AHSP*** | 1.65 | 1.27 |
| 50 | ***C8orf38*** | 1.62 | 1.61 |
| 51 | ***BTNL8*** | 1.51 | 1.42 |
| 52 | ***PHOSPHO1*** | 1.48 | 1.21 |
| 53 | ***FCER1G*** | 1.48 | 1.21 |
| 54 | ***CMTM2*** | 1.47 | 1.60 |
| 55 | ***LRG1*** | 1.42 | 1.72 |
| 56 | ***CXCR2*** | 1.41 | 1.32 |
| 57 | ***HBD*** | 1.40 | 1.38 |
| 58 | ***BAX*** | 1.39 | 1.98 |
| 59 | ***ANXA3*** | 1.37 | 2.04 |
| 60 | ***HLA-DQA2*** | 1.30 | 1.51 |
| 61 | ***PI4KA*** | 1.28 | 1.22 |
| 62 | ***MGAM*** | 1.27 | 1.25 |
| 63 | ***CHI3L1*** | 1.26 | 2.35 |
| 64 | ***TNFRSF10C*** | 1.25 | 1.22 |
| 65 | ***MPO*** | 1.23 | 1.43 |
| 66 | ***DEFA4*** | 1.22 | 2.00 |
| 67 | ***FAM177B*** | 1.21 | 1.22 |
| 68 | ***MMP8*** | 1.20 | 2.65 |
| 69 | ***MT2A*** | -1.25 | -1.53 |
| 70 | ***MTRNR2L1*** | -1.34 | -1.39 |
| 71 | ***SNAI1*** | -1.67 | -1.26 |
| 72 | ***IGLL3P*** | -4.35 | -5.57 |
| 73 | ***RBP1*** | -7.06 | -7.06 |
